# Factors Worsening and Mitigating the Consequences of the COVID-19 Outbreak on the Overall Health of Informal Caregivers of Older People with Long-Term Care Needs Living in Germany and in Italy

**DOI:** 10.3390/ijerph19031694

**Published:** 2022-02-01

**Authors:** Sara Santini, Marco Socci, Paolo Fabbietti, Giovanni Lamura, Andrea Teti

**Affiliations:** 1 Centre for Socio-Economic Research on Aging, IRCCS INRCA-National Institute of Health and Science on Aging, Via Santa Margherita 5, 60124 Ancona, Italy; s.santini2@inrca.it (S.S.); g.lamura@inrca.it (G.L.); 2 Laboratory of Geriatric Pharmacoepidemiology, IRCCS INRCA-National Institute of Health and Science on Aging, Via Santa Margherita 5, 60124 Ancona, Italy; p.fabbietti@inrca.it; 3 Institute for Gerontology, University of Vechta, 49377 Vechta, Germany; andrea.teti@uni-vechta.de

**Keywords:** COVID-19 outbreak, elder care, Germany, health, informal caregivers, Italy, Long-Term Care systems, older people, formal care services

## Abstract

Population ageing and the higher prevalence of multimorbidity in later life are increasing the demand for Long-Term Care (LTC) worldwide; this has been exacerbated by the COVID-19 pandemic. As in Europe and beyond, the bulk of care for frail older people is carried out by informal caregivers. This study aimed at understanding the factors affecting the overall worsening health of informal caregivers of older people with LTC needs living in Germany and Italy during the outbreak. To this purpose, 319 informal caregivers (149 in Germany and 173 in Italy) were surveyed online in 2020–2021. A logistic regression analysis was performed by country, to obtain an adjusted estimate of the risk of worsening of caregivers’ health. This risk increased by 42% for German caregivers compared to Italian ones, despite the former receiving more formal services. This may depend on different quality standards of LTC services and caregivers’ expectations, and on differing policies concerning migrant care workers (MCWs) during the outbreak, who could not enter Germany and were “trapped” at care recipients’ homes in Italy. Results call for in-home care reforms and policies guaranteeing more effective caregiver support, home care services and fairer working condition for MCWs in both countries.

## 1. Introduction

### 1.1. The Mismatch between the Demand and Provision of Formal Long-Term Care Services for Older People with Disabilities in Germany and in Italy

As a consequence of population ageing, the demand for Long-Term Care (LTC) (i.e., the delivery of a range of care services to meet the health needs of people limited in their ability to live independently) is dramatically increasing across the world, threatening LTC systems’ sustainability. This is especially true for those countries with older age structures, like Germany and Italy, the focus of the study presented here, with 21.8% and 23.2% of their population aged 65 and over [1].

Although Germany and Italy are characterized by a similar ageing processes, the response to the growing need for care differs in these countries, in terms of funding and service provision, due to different LTC concepts. Germany has one of the most elaborated and all-embracing welfare regimes, characterized by universal public and free health care, including monetary transfer, LTC facilities and home health care (as detailed below). Care, medication and nursing are funded by an obligatory state insurance system [2], introduced in 1995 by a national law as compulsory insurance to provide social security for those needing LTC. It provides medium-level support to dependent older people, emphasizing family, home and outpatient care, complemented by publicly financed services [3,4]. This monetary care contribution is calculated according to individually determined care degrees (1–5), ranging from 40 EUR to 250 EUR in the case of a low care needs (care degree 1), up to almost 2000 EUR in a situation of complete care needs in a nursing home (care degree 5) [5].

In Italy, characterized by a Mediterranean welfare regime [6], LTC is not conceived and organized as a comprehensive model. However, it juxtaposes multiple legislative interventions to integrate different national and regional legislation and social and health services [7]. Italian LTC provides a medium/low level of support, with unregulated cash payments and service delivery [4]. It stands on three pillars: residential care, home care and monetary transfers.

The residential care (i.e., nursing homes and day care services) is managed mainly by private bodies or Non-Governmental Organisations, acting as third parties of municipalities [8,9]. The provision of beds in nursing homes differs region by region, but it remains far from addressing the older population’s needs, as specified below.

Home health care services (e.g., medications, physiotherapy, blood tests) are delivered by the regional health care systems/authorities, while municipalities provide social care services (e.g., meals preparation and distribution). However, the delivery of home care services is scarce [10]: in 2016, on average, just 16 h of public home care services were provided per older person in total in one year [11].

The most common monetary transfer is the State Care Allowance “*indennità di accompagnamento*” in Italian) [12], introduced by Law number 18/1980, provided by the National Social Security Institute (*Istituto Nazionale di Previdenza Sociale-INPS*) and financed by tax revenues. It is a fixed monthly contribution (cash-for-care) of 522,10 EUR per month, provided to dependent people, regardless of the degree and type of care needed and economic situation of beneficiaries, without the obligation to declare the use made of it [12].

Given that in Germany as well as in Italy, the LTC systems are conceived as a support complementary to the main one provided by family members, in both countries there is a mismatch between LTC service demand and delivery, though to different extents.

In Germany, in 2014, the prevalence of multimorbidity in the 65+ age group was between 64% and 84% (men: 60% and women: 81%) [13]. In 2019, the number of people in need of care totalled 4.1 million, of whom 80% were cared for at home. The remaining 20% were cared for in one of the 15,400 LTC facilities (e.g., nursing homes) [14]. Older people with nursing care needs can choose between professional service (*Pflegesachleistung*) or monetary transfers. In the first case, older people are taken care of at home by professional caregivers who have a contract with nursing insurance funds and nursing homes [15] and provide a non-standardised number of hours of care in accordance with different degrees of care need. Alternatively, elder home care can be supported by cash benefits, with the prerequisite that older people have to be taken care of at home by their relatives, who are entitled to receive the allowance paid by the nursing insurance fund. It is also possible to combine cash benefits and professional home health care [16,17].

In Italy, in 2019, 3.8 million people aged 65 and over (28.4% of this population group) had severe difficulties in basic functional activities (i.e., severe motor, sensory and cognitive limitations), and more than one in two older people had multimorbidity, reporting at least three chronic diseases [18,19]. Of these, 90% were cared for at home (compared to 80% in Germany) and 10% lived in one of the 7372 nursing homes located in the country (about half of those available in Germany), whose number of beds differs region by region, ranging from 25 per 100 not-self-sufficient over-75 people in Trentino-Alto Adige (in Northern Italy) to 0.65 in Basilicata (Southern Italy) [20].

Thus, in comparison, the percentage of older people with needs accommodated in LTC facilities in Germany is twice as high as in Italy (20% and 10%, respectively).

### 1.2. Informal Caregivers in German and Italian LTC Systems

LTC systems in European countries are dictated by specific policy lines influencing the level and modalities of state support and the nationally prevalent care models, including the distribution between formal and informal care provision [3]. Regardless of the level of development of the LTC systems and of the welfare regime, informal caregivers play a pivotal role in the provision of assistance to older and/or disabled family members across Europe and beyond [21]. While country-specific estimations of the rate of informal caregiving are often lacking [22], a comparison based on three European Surveys—the European Health Interview Survey (EHIS), the European Quality of Life Survey (EQLS), and the Study on Health and Ageing in Europe (SHARE)—show in both countries examined similar prevalence rates of informal caregiving (albeit different estimations due to partially different methodological aspects characterizing the three surveys): 17.70% of Italian adults aged 50 and over (SHARE: 13.66/EHIS: 20.02/EQLS: 19.40) and 18.26% (SHARE: 23.80/EHIS: 20.32/EQLS: 10.66) for the German population in the same age group [23].

Strong differences emerge with regard to gender. In Italy and in Germany the share of women aged 45–64 providing informal care is 27% and 11%, respectively, compared to 18% and 8% of men in the same age range. As for intensity of care, Italian women spend, on average, more than 17 h per week providing care, compared to about 12 h provided by men. The situation is reversed in Germany, where men carry out about 11 h of care per week, and women a little more than 10 [24].

A similarly differentiated picture concerns the estimated economic time value of informal care. In Italy, this value was estimated to correspond to 3.6% of the GDP vs 0.9% of the GDP expenditure for formal care, while in Germany the economic value of informal care reaches only 0.9% of GDP, compared to 2.1% of the GDP for formal care, thus showing that formal care expenditure in Germany is double the Italian one [21].

### 1.3. Migrant Care Workers in German and Italian LTC Systems

Despite the described differences, German and Italian LTC systems are similar in another aspect, in addition to the similar presence of 50+ informal caregivers (as described above), i.e., the extensive employment of Migrant Care Workers (MCWs) as a response to the absence of an appropriate and sufficient supply of residential and home care service workers.

About 200,000 MCWs from Central and Eastern Europe are employed in Germany, but the estimated number of unregistered care staff is high [22] this putting MCWs at risk nor of having fair and equal working condition neither of accessing social protection and training that, conversely, are included in the fifth social pillar foreseen by the European Commission [25]. The live-in care model in Germany is based primarily on female workers from Poland, Romania and Slovakia, providing home care for one or more older people. Typically, two (or more) commuting MCWs alternate shifts of 2–12 weeks [26]. In this timeframe, they live 24/7 with the older care recipient.

In Italy, the number of families employing MCWs seems to be higher, with this facilitated by the lack of any restrictions in the use of the State Care Allowance. Since it is not mandatory to justify how and for what it is spent, the latter is often used by beneficiaries and their family members to pay the MCWs. There were almost one million declared domestic workers in Italy in 2020: 90% were female, and 70% had a migration background, mainly from Eastern Europe [27]. It is estimated that 60% of Italy’s domestic workers are undeclared (i.e., without a formal employment contract), which means that around 1.3 million people have no rights in the workplace [25]. Therefore, MCWs represent, together with family members, the informal side of the LTC system for the older people living in Italy [28]. They are the main means to guarantee tailored and around-the-clock assistance and supervision to older people with LTC needs, by helping family members hold on to the tradition of family care for ageing parents/relatives.

The main difference with the German model lies in the fact that MCWs in Germany are recruited and engaged by brokering agencies that also organize the travel to Germany. Conversely, in Italy, MCWs are mainly recruited by older people’s families via word-of-mouth and NGOs (without brokering agencies) and employed mostly in live-in situations and by long-term work commitments (according to their long-term migration plan), even if the frequent turnover of the workers is quite common, given the 24-h caring activities that they are required to carry out.

### 1.4. The Impact of COVID-19 Containment Measures on Informal Caregivers’ Overall Health

Previous research has demonstrated that the negative aspects of informal caregiving to older relatives are associated with depression, and very often informal caregivers can experience high levels of anxiety, stress, morbidity, physical problems and low quality of life (especially when they take care of older relatives with dementia) [29,30]. Caregiving responsibilities (e.g., moving, toileting, feeding, cleaning the care recipient and administrating drugs) can represent a risk for informal caregivers’ overall health (i.e., physical and mental well-being) [31,32,33,34,35] that can vary with caregivers’ health conditions [36] and may worsen, especially when the caregiver is cohabiting with the care recipient [37]. Caregiving can also lead to difficulties in social life and in labour market participation, in reconciling paid work and care duties [38,39,40] and in social inclusion and participation [41].

Such conditions have been worsened by the COVID-19 outbreak. The latter has entailed the cancellation and/or the postponement of many social and health care services targeted to older people, e.g., day care centres and home care services, the adoption of contingency measures for nursing care homes (e.g., relatives’ visit interruptions) and a range of restrictions affecting social life (better known as “stay at home measures”).

The latter exacerbated situations of social isolation and worsening mental health, already evident among informal caregivers before the pandemic, especially among those with limited technological skills, as they cannot benefit from the extensive opportunities to connect with others via the internet or social media [42].

The Government Responses Stringency Index (GRSI) [43], collecting systematic information on policy measures that governments have taken to tackle COVID-19, has similar scores for the German and the Italian government responses, presenting a maximum difference of three points over the outbreak, except for the periods around 21 March 2020, when the government response stringency level was 68.06 in Germany and 91.67 in Italy; on 21 March 2021, when it was 75 and 84.26, and on 17 September 2021, when it was 56 and 68.98 in Germany and in Italy, respectively. Moreover, according to the indicator of the GRSI entitled “Protection of elderly people”, German as well as Italian governments adopted “extensive restrictions for isolation and hygiene in Long-Term Care facilities, by prohibiting all non-essential external visitors, and/or requiring all community dwelling older people to stay at home and not leave the home with minimal exceptions, and receive no external visitors”.

The interruption, postponement and cancellation of social, health and community services for older people with LTC needs and for informal caregivers made the latter feel alone [44] and entailed the increase of prevalence and intensity, or the change, of caring activities carried out by informal caregivers [45], with a dramatic impact on their mental health and well-being [46,47,48,49,50,51,52,53]. Evidence suggests that the pandemic dramatically impacted the daily life of informal caregivers. In many countries, due to both the shortfall in support services and the persistent lack of information and guidance on the appropriate behaviour to adopt, informal caregivers experienced a great increase in their psychological and physical burden [54] as well as stress and anxiety [55]. This is also the case for Italy and Germany, as highlighted by recent studies [52,56].

Moreover, lockdowns put in place in both countries caused a shortfall in private care services, such as those provided by MCWs. In Germany, the closure of borders during the peak of the infection, namely between March and June 2020, interrupted the flow of MCWs from Eastern European countries, causing an increase of informal caregiver efforts [49,57]. Nevertheless, the German government extended MCWs’ visas and put in place incentives for keeping MCWs in the country, e.g., extending workers’ shifts and re-establishing unofficial transnational mobility for MCWs; German border authorities have been reported to have been permissive in letting care workers cross the borders [58].

In Italy, during the first wave of the pandemic, MCWs living in the same households as older people were considered as cohabiting family members, while the work of non-cohabiting MCWs was subject to the restrictions provided for all citizens who travelled to reach the workplace, i.e., self-certification indicating the work place address and personal details of the employer (i.e., the older care recipients or their relatives). Thus, live-in MCWs continued living with older care recipients, but in a condition of isolation, because often older people’s relatives lived out of the municipality and could not reach them due to pandemic-related mobility restrictions. Conversely, MCWs who did not reside with their charges often lost their job because they were infected with the virus or due to the older person’s relatives’ fears of transmission.

In light of the above, informal caregivers saw diminishing care supports, both formal and informal, during the pandemic. The first, due to the interruption of home care and day care services, and the second due to the fear of contagion and of sanctions and fines imposed by the physical distancing rules that led people to decrease social contacts and visits to frail and disabled older relatives.

Recent studies [48,59] have highlighted several stressors for informal caregivers, in addition to those that could be experienced by the general population as a consequence of the COVID-19 outbreak, as emotional stress, health stress (due to the reduced access to health care services during the pandemic) [60] and social isolation. Many informal caregivers reported worse health conditions and quality of life as a consequence of the outbreak in different European countries [45,51,61], and evidence shows that this occurred more strongly than among non-caregivers [62].

Despite these findings, the impact of pandemic-related interventions and policy measures on home care and community-based care settings is still rather neglected by the literature, especially with regard to their effects on informal caregivers of older people with LTC needs.

### 1.5. Aim of the Study

In light of the above, this study aims at advancing the available knowledge on the impact of the COVID-19 outbreak on the perceived overall health of informal caregivers of older people with LTC needs living in Germany and in Italy. In fact, to the best of our knowledge, there are no other studies analysing the impact of the COVID-19 outbreak on informal caregivers’ health in these two countries, having different LTC systems. This is an important topic to explore, even in light of the evidence available in the literature concerning the negative outcomes on informal caregivers’ health in non-pandemic times.

These two countries belong to two different welfare systems according to the classification of Esping-Andersen [6] and have been recently assigned to two different clusters by Tur-Sinai et al. (2021) [63]. This research analysed the response of LTC systems to the challenges posed by the pandemic and showed that Germany demonstrated high resilience in terms of both informal care (i.e., that provided by children, other relatives and non-relatives, like neighbours) and formal home-based care services, while Italy was characterised by a medium-low level of resilience, with weaker support by both informal care networks and formal home care [63]. The latter study stemmed from the hypothesis that caregivers’ health could have been influenced by the decrease/cancellation/postponement of social and health care services because of the outbreak and by the peculiarities of the LTC system characterizing the two countries.

In order to verify this hypothesis, the study aims at answering the following research questions: (1) What was the impact of the COVID-19 outbreak and of the consequent reduction of social and health care services on the overall perceived health status of informal caregivers of older people with LTC needs living in Germany and in Italy? (2) What are the predictors of a decrease in informal caregivers’ overall perceived health status and what are the mitigating factors?

## 2. Materials and Methods

The study is based on an online survey targeting European informal (i.e., unpaid) caregivers providing regular care and/or support (i.e., not occasional or temporary) to one or more people with their daily activities, personal care or in any other way due to their physical or mental illness, disability or old age. MCWs are not included in the sample because they receive an income and have a regular formal work contract.

The survey, carried out between November 2020 and March 2021 and promoted by Eurocarers, was available in 10 European languages (i.e., Czech, English, Estonian, Finnish, Finnish/Swedish, French, German, Italian, Portuguese, Swedish) [53].

Both in Italy and Germany, the online questionnaire was disseminated by the Eurocarers’ broad member organisations network. Respondents were mainly recruited by means of dissemination activities carried out through websites, social media channels (mainly on Twitter and Facebook groups or pages targeting informal caregivers and cared-for persons), local charities, webinars, and welfare or voluntary organizations at national, regional, or local levels. These procedures were repeated regularly in the period between November 2020 and March 2021.

This study, which reached an overall sample of 2468 European caregivers from 16 countries, is focused on two sub-samples of 146 informal caregivers living in Germany and 173 in Italy, providing care to older people aged 65 and over having LTC needs (i.e., experiencing multiple chronic diseases limiting their activities of daily living). Since several studies have analysed the consequences of the first pandemic wave in Spring 2020 [46,47,48,49,50,51,52,59,60,61,62,63], an added value of the study is that it provides evidence about the impact of the COVID-19 outbreak in winter 2020–2021, a critical period in which the effects of the first and following pandemic waves in Europe may have cumulated over time.

Electronic consent was requested from respondents before filling in the questionnaire, confirming that participants (1) had read the background information to the study; (2) voluntarily agreed to participate; (3) were at least 18 years old. All responses to the survey were collected anonymously, in compliance with the EU Regulation No. 679 of the European Parliament and of the Council of 27 April 2016 and the Helsinki Declaration (2013).

### 2.1. Data Collection Tool and Variables

The questionnaire included multiple choice questions and moved through six thematic areas: (1) caregivers and care recipients’ socio-demographic information; impact of COVID-19 on (2) the caregivers’ health status and on the caring situation; (3) the availability and use of social and health services both for caregivers and for care recipients; (4) caregivers’ employment and economic status; (5) the use of technology during the outbreak; (6) caregivers’ suggestions on how to improve social and health care services for better supporting them during the outbreak and beyond.

The study outcome variable is the response to the question “Considering your current situation, compared to before the pandemic, how has the pandemic impacted your overall health status?”. Respondents had three options for answering: “my overall health status… (1) improved; (2) did not change; (3) worsened”. We dichotomized this variable into “My overall health status improved/did not change” vs. “My overall health status worsened”.

Several variables were used for the analysis. The variable entitled “Caregiving (difference post–pre in hours of care)” indicates the difference between the hours of care provided before and during the outbreak (i.e., at time of answering the questionnaire).

The variable “Formal service provision (Discontinuity)” was calculated for highlighting whether there was at least one “yes” to the items “Social and health services decreased”, “Social and health services postponed” or “Social and health services cancelled”.

The variable “Service provision (Continuity)” was calculated for highlighting whether there was at least one “yes” to the items “Services adapted (e.g., online/telephone health consulting, etc.)”, “Services continued as before” or “Services increased”.

The variable “Formal support effectiveness” indicates at least one “very/extremely effective” service among the options “General Practitioner”, “Public health services/professionals”, “Public social services/professionals”, “Private care services/providers”, “The carer/patient organisation(s) with which I am in contact” and “Pharmacies”.

The variable “Informal support effectiveness” indicates at least one “very/extremely effective” support among the options “Church/Religious organisations”, “Voluntary organizations”, “Family members”, “Friends/Neighbours”, “Migrant/private care workers”.

### 2.2. Statistical Analysis

Informal caregivers and care recipients’ characteristics, divided by country, were compared using the chi-square test for categorical variables and the Students t-test for continuous variables. Data are reported as means (±SD) for continuous variables and as absolute frequencies for categorical variables. Thus, we compared subjects according to the outcome variable “overall health status” (improved/did not change vs. worsened) and to the variable “country” (Germany vs. Italy).

In order to obtain an adjusted estimate of the risk of worsening in overall health status, a logistic regression analysis was performed by country. Initially, we considered only crude models, but then we adjusted for age and gender, and finally we built a fully adjusted model, in which there were only the significant variables. A *p*-value < 0.05 was considered statistically significant. Statistical analysis was carried out using SPSS for Win V24.0 (SPSS Inc., Chicago, IL, USA).

## 3. Results

### 3.1. Informal Caregivers’ Characteristics and Living Arrangements in Relation to the Care Recipient

The questionnaire was answered by 146 informal caregivers of older people from Germany and 173 from Italy (319 family caregivers in total). In both countries, respondents were mostly females (89% in Germany and 78% in Italy), and the mean age was about 55 years. Italian caregivers had a (statistically significant) higher educational level compared to German ones. In fact, Italian respondents had mostly upper secondary (high school degree) (44%) and tertiary education (bachelor’s degree) (44%), with only about 7% having lower secondary education (secondary school degree), while German caregivers had mainly lower (26.7%) and upper secondary educational levels (26%) (Table 1).

Considering the relationship with the care recipients, despite some country differences, though non-statistically significant, about 71% of the overall sample was taking care of grandparents and parents (including in-laws), and more than 18% was looking after spouses.

### 3.2. Care Recipients’ Description

The majority of care recipients were aged over 80 and female, both in Germany (58%) and—with a higher percentage—in Italy (72%) (Table 1). In Germany, 79% suffered from physical disability, and in Italy more than 67%. In Germany 68.4% and in Italy 61.3% of older care recipients also reported psychological or mental issues, such as depression or anxiety (Table 1). In both countries, about 7 older care recipients out of 10 had cognitive impairment (i.e., dementia and memory loss problems), and about 3 out of 10 had neurological disabilities (other than dementia or memory problems). Finally, more than 75% of care recipients in Germany and 63% in Italy suffered from chronic illness (e.g., diabetes and cancer) and 68.7% and 48.8% in Germany and in Italy, respectively, from other long-term health conditions. Thus, the multimorbidity of care recipients is a widespread critical issue in both sub-samples.

### 3.3. Living Arrangement and Caregiving Intensity

Out of 319 respondents, more than 68% lived in the same household or in the same building. Almost 17% of Italian and more than 9% of German respondents lived within walking distance to the cared-for person, while about 36% of German caregivers lived further than walking distance, compared to about 27% in Italy. This suggests that distanced caregiving was more common in Germany than in Italy.

German respondents provided 32.4 h of care per week on average before the outbreak and 44.1 during the pandemic. Italian caregivers provided about 40.7 h of care per week before the outbreak and 46.8 during.

Finally, German caregivers of older people with severe health conditions were more numerous than Italian caregivers, and the difference is statistically significant, namely for caregivers of relatives with physical disabilities (*p* = 0.024) and chronic illnesses (*p* = 0.020).

### 3.4. Formal Support Services Received during the COVID-19 Outbreak

Informal caregivers living in Germany received more formal support than Italians (Table 2). The amount of several basic services was similar in the two countries: e.g., health care support was reported by about 54% of German carers and more than 44% of Italian caregivers; education by more than 11% in both countries; and financial support by more than 16% of the German sub-sample and 10% of the Italian one. However, overall, German informal caregivers received double or even triple the formal support compared to Italians, with a statistically significant difference. In fact, social care services were received by about 30% of German vs. 15% of Italian respondents; face-to face help by 29% of German vs. 6% of Italian respondents; practical help by 35% of German vs. 17.4% of Italian respondents.

Moreover, about 34% of German informal caregivers could count on grocery and meal delivery at home vs. 14% of Italians, and 44% of German respondents benefited from medication and drug delivery at home vs. about 18% of Italians. Furthermore, the number of German caregivers who used transportation services was triple compared to the number of Italian caregivers (about 31% in Germany vs. 11% in Italy), and respite care/relief services were received by more than 24% of German respondents compared to about 11% of Italian ones.

German caregivers found several services more effective than Italian caregivers, namely public health services (17% in Germany vs. 10% in Italy), social services (13% in Germany vs. 5% in Italy) and private care services (27% in Germany vs. 20% in Italy). The supports received by family, friends and neighbours, GPs, pharmacies, and carer/patient and church/religious organisations were considered as more effective by German caregivers than by Italian ones. Conversely, the Italian respondents considered the help of MCWs about eight times more effective than German informal caregivers (about 28% in Italy vs. 3.5% in Germany), as well as the help received by voluntary associations (10% vs. 1% in Germany). These differences between German and the Italian caregivers were almost always statistically significant (Table 3).

Table 4 shows that 35% of Italian informal caregivers experienced difficulties for themselves “always” or “often” vs. 22.4% of German ones. This finding is mirrored by different levels of feeling overwhelmed due to the outbreak. In fact, about 58% of Italian caregivers strongly or quite agreed on this feeling vs. 36.5% of German respondents. However, about 32% of German respondents felt unable to look after their own health and well-being vs. about 14% of Italian informal caregivers, showing in this case a different picture.

### 3.5. The Impact of the COVID-19 Outbreak on Informal Caregivers’ Overall Health

There is no correlation between caregivers’ age, gender, educational level and worsened health condition in the two countries, albeit the mean age of caregivers reporting health worsening was higher than that of caregivers reporting no change in their health condition due to the COVID-19 pandemic (Table 5).

Data on the increase of caregiving hours show that in both countries the share of informal caregivers reporting a worsened health status is about the same of those who reported an increase in the number of hours spent in caregiving: +13.9 h among the German respondents and +10.7 among the Italians.

Table 5 shows that in Italy 91% of informal caregivers reporting a worsened health condition saw social and health care services cancelled/interrupted/postponed during the COVID-19 pandemic. The difference between the caregivers reporting worsened health status and those who did not report any change in their overall health is statistically significant (*p* = 0.032).

Concerning formal support (i.e., public and/or private help received by health care professionals), a polarised picture emerges with regard to the difference observed between the group of informal caregivers reporting worsened health and those with an unchanged health status (albeit not statistically significant). In fact, in Germany, formal support was received by 76.7% of informal caregivers without health condition worsening and by 68.2% of informal caregivers with health condition worsening. On the contrary, in Italy, formal support was received by 59.1% of respondents not reporting worsened health and by 64.6% of respondents reporting it.

In Germany, there is a correlation between the unchanged health condition and the availability of extremely useful informal supports. In fact, in this country, 70% of informal caregivers who did not report poorest health could count on informal supports. The difference with the group of informal caregivers reporting worsened health is statistically significant (*p* = 0.013). Thus, in Germany, receiving informal support during COVID-19 positively impacted the informal caregivers’ overall health.

Both in Germany and Italy, more than 60% of caregivers reporting a worsened health condition lived with the older care recipients (62.4% in Germany and 64.1% in Italy). Few informal caregivers were infected with the virus, and no correlation was found between the infection and the worsened health condition.

Both in Germany and Italy, caring for older care recipients with psychological/mental health issues (e.g., depression, anxiety, etc.) increased the chance of worsened health condition. In fact, older care recipients with this kind of disease are more frequent in worsened health caregiver groups with respect to the improved/unchanged group (*p* = 0.001 in Germany and *p* = 0.025 in Italy). In Italy, older care recipients with neurological disabilities or learning difficulties are more frequent in worsened health caregiver groups with respect to the improved/unchanged group (*p* = 0.027).

### 3.6. Predictors and Protective Factors of Informal Caregivers’ Overall Health Worsening

The results of the logistic regression analysis concerning predictors of informal caregivers’ change in health status are reported in Table 6. Here, the “fully adjusted” model reports only variables achieving statistical significance in the age and gender adjusted models. In this section, we focus our attention mainly on the statistically significant variables.

Looking at the country level, in Germany, receiving informal support mitigated the risk of overall health worsening (OR = 0.45), while psychological/mental health issues of older care recipients (e.g., depression, anxiety, etc.) increased the risk (OR = 3.01). Living within one hour travelling time to the older care recipient emerged as a possible protective factor for overall health worsening (OR = 0.39 in the adjusted model), albeit with no statistical significance.

In Italy, discontinuity (i.e., interruption/postponement/cancellation) in formal social and health care service provision was a predictor of the overall health worsening for informal caregivers, being associated to an increase of this risk by more than 2.5-fold on average (OR = 2.54). Moreover, in Italy, living at a walking distance seemed to prevent informal caregivers’ health deterioration (OR = 0.40 in the adjusted model), unlike what was found in Germany, where living at a walking distance seemed to contribute to an increase in the risk to the caregivers’ health condition (OR = 0.69 in the adjusted model), albeit with no statistical significance.

## 4. Discussion

This is one of the few cross-national studies focusing on the impact of the COVID-19 outbreak on the health condition of informal caregivers of older people with LTC needs living in different LTC systems and involving large samples of informal caregivers, and carried out during the critical pandemic phase of winter 2020–2021. As such, it can provide useful insight into the effect of macro-level factors (such as those related to the overall functioning of and support provided by the welfare system at the country level) on the individual (i.e., micro-level) condition of one of the main pillars of our LTC systems: informal caregivers.

Understanding such an influence can be important because, in the last decade, we have witnessed the tendency of European families (at the micro-level) to increase in-home care for the most vulnerable older people, in order to ensure a better quality of assistance and to reduce the costs of institutionalization [64]. This trend is mirrored at the macro-level by the EU policy agenda (especially the European Pillar of Social Rights) [65], which considers community care as a priority in the formal health care sector in the last few years and home-based care as a practical measure to contain the costs of services and address older people’s preferences. All of this puts more and more pressure on informal caregivers.

The reported findings confirm the increase in the intensity of care for both German and Italian informal caregivers [44] and the worsening of their overall health as a consequence of the pandemic [51], as already highlighted by several, primarily single-country, studies [45,46,47,48,49,50,51,52,53,54,55,56].

The main novelty of this study consists of showing that predictive informal caregivers’ health worsening and mitigating factors differ in the two countries. In fact, the interruption/cancellation/postponement of formal social and health care services increased the risk of health worsening only in Italy, where the discontinuity of service provision, more severe than in Germany, was not counteracted and balanced by informal care support provided by other family members different from the main informal caregiver and/or friends and neighbours. Conversely, German caregivers could count on both sufficient (despite reduced) formal social and health care services and on a still relatively substantial informal care support.

The lack of informal support in Italy can be explained by the severe “stay at home” measures (accompanied by a massive media campaign) that were instituted in the country for protecting older relatives from the infection and that weakened family, friend and neighbourhood relationships, or at least prevented them from translating into effective help.

It is worth noting that the risk of health worsening increased by 42% for German caregivers compared to the Italian ones. This finding is surprising, considering that the German informal caregivers received two to three times the formal health and social services of the Italians (e.g., social care services, face-to face help groups, meal delivery, respite care) during the pandemic. Nevertheless, one explanation can certainly lie in the higher number of care recipients with long-term health conditions found in Germany (68.7%) compared to Italy (48.8%) and on the worsened mental and physical health condition of the German care recipients. In fact, psychological/mental health issues increased threefold compared to the risk of caregivers’ physical health deterioration within the German sample.

However, the higher risk of health worsening in the German sample may also depend on two additional factors. The first lies in the difference between the two LTC systems and in the related expectations concerning formal support in the two countries. The German LTC system stands on the provision of a large set of formal home-based in-kind services in normal times, which were probably not provided at the same standards during the pandemic. Thus, the German informal caregivers, who are used to being widely supported by formal health and social care services, had to provide 8.7 h of care per week more than before the pandemic, and consequently they had a stronger self-perception of the impact of the shortfall in services on their well-being than Italian caregivers, despite the fact that they received more formal services than the Italian caregivers during the outbreak. The latter, conversely, very seldom receive extensive in-kind home services (even before the healthcare crisis). Therefore, the vast majority did not expect to receive a wide spectrum of homecare services during the lockdown and thus did not perceive that their health condition was worsened during the pandemic, despite feeling overwhelmed.

The second health worsening factor for the German informal caregivers may lie in the different regulation of the care work provided by MCWs in the two countries. In Germany, MCWs usually work for shorter periods of time (e.g., two weeks) and then alternate with other migrants, substituting “shifts” (transportation included) regulated by brokering agencies. The closure of borders in early Spring 2020 limited the entry of MCWs from Poland, Romania and Slovakia, thus depriving informal caregivers of this “semi-formal” support. Later on, the flow of MCWs was allowed again, but not to an extent that could cover the increasing and unprecedented demand for care coming from frail older people in Germany [65].

Conversely, in Italy the presence of MCWs with a long-term migration project and the lack of any kind of regulation limiting their access and work in older people’s homes allowed Italian caregivers to be able to count on their help. In fact, in this country, many MCWs remained at home with the care recipient, bearing the greater burden of assistance in place of relatives, who could only occasionally reach older parents due to the restrictions, which during the first lockdown in early Spring 2020, allowed travel only within the neighbourhood (and only for cases of proven need) within the municipality of a residence [66,67,68]. This interpretation is confirmed by the findings on the effectiveness of the received services in the two countries. In Italy, in fact, MCWs represented the third-most effective support received during the pandemic (after pharmacies and other family members), while in Germany they were considered only as the second-last one.

Thus, the study confirms the lower resilience of the Italian formal and informal care supports in response to the reduction of the care services during the pandemic compared to Germany [63]. In addition, this study identifies MCWs as one of the main sources of resilience in the German and Italian LTC systems and advances further hypotheses for the interpretation of the results coming from previous and future studies focusing on the response of the LTC systems to the challenges posed by the COVID-19 health crisis.

### 4.1. Suggestions for Research and Policy

This study shows that a remarkable share (almost two out of three) of informal caregivers in Italy and in Germany felt overwhelmed during the last pandemic wave, pointing to a lack of appropriate supports. These findings suggest the need for further qualitative studies on the in-depth nuances of this phenomenon, in order to integrate past theoretical and conceptual frameworks in this field with more robust evidence on how to best support informal caregivers in emergency situations.

During the COVID-19 pandemic, the shortfall in health and social care services, and especially the lack of integration between formal and informal supports and between social and health care services for both older people and informal caregivers, weakened and threatened the effectiveness of LTC systems to respond to the challenges posed by the pandemic in the two investigated countries and had an impact on the health of informal caregivers, especially in Italy. This finding calls for a better and systematic involvement of informal caregivers in the planning of care interventions and services targeted to older people with LTC needs, especially those with physical and mental health issues, and their caregivers, as well as in the design of research efforts in this area. This entails, especially in Italy, the recognition of the role of informal caregivers both from the legislative point of view, through a national law and coordinated, comprehensive supporting policies (as it partly occurs in some Regions, e.g., Emilia-Romagna) and from a social security perspective, with the recognition of the time spent in informal care counting as pension credits or of care experience and skills earning qualifications for a possible later inclusion in the labour market (e.g., in the elder care sector).

The pandemic shed light on the need for improving home care services targeted to older people in need of LTC in both the countries. In fact, in Germany, the demand for home care during the lockdown was covered almost entirely by informal caregivers (i.e., family members, neighbours and friends), while in Italy, it was covered almost exclusively by relatives with the support of MCWs. In Italy, the latter guaranteed both flexibility and continuity in home care provision during the pandemic, continuing the suboptimal care system characterising this country [69,70].

This study underlined once again that the pandemic has brought to light the lack of sustainability and social justice of LTC systems based on the extensive contribution of MCWs in elder care. The support from MCWs has certainly benefited Italian informal caregivers, but it is conceivable that it has led to an increase in burden and stress for the MCWs, who even in non-pandemic times are exposed to a large number of working hours and receive low wages, often outside of any formal working contracts and social security coverage. In contrast, in Germany, the unavailability of MCWs due to the closure of the borders sent the German elder care system into turmoil and considerably increased the care burden of informal caregivers.

In light of this, it is recommendable to systematically consider MCWs and family caregivers of older adults as essential components of the LTC system, especially in those countries where their contribution is more substantial, and search for solutions allowing them to be routinely included in the planning and implementation of care provision.

From a policy perspective, European health workforce governance should be developed, which connects health and social care system needs, health care, labour markets and MCWs, both at a macro and at an individual level, in order to create policies that guarantee the continuing provision of home care as well as fair working conditions for MCWs in a “win–win” perspective. The European Commission and the Member States have indeed recognised and defined MCWs as “key-workers”, because they carried out essential functions during the pandemic to keep European citizens healthy, safe and fed [71,72]. MCWs were also defined as more vulnerable than native care workers, because they were more frequently employed under temporary contracts, earned lower wages and could not work from home due to the typology of their job (i.e., personal home care), thus exposing MCWs, often working in more than one household, to a higher risk of infection [73].

In Germany, therefore, a reform of the live-in care market is required to regulate the work of the brokering agencies and ensure fair and better working conditions and social security measures for MCWs and the continuity of care to older care recipients and informal caregivers. Similarly, in Italy, stronger measures have to be put in place for hindering the irregular employment of MCWs and guaranteeing fair working conditions.

Moreover, in Italy, where this study highlighted the weakening of family and community ties, policies and initiatives targeted to restore and strengthen intergenerational and intra-generational family, friend and neighbourhood relationships interrupted during the pandemic seem to have become a priority for weaving and mending the informal support network at the community level, namely in the face of an inefficacious formal support system.

Furthermore, the integration of multiple types of formal and informal care solutions can simultaneously respond not to only the health but also to the social and emotional needs of both informal caregivers and older care recipients, especially in the face of a complex care situation like the one represented by the global pandemic crisis.

### 4.2. Study Limitations

Despite the innovative evidence highlighted by this study, some limitations should be taken into account when considering these findings.

The first limitation concerns the rather small sample size on which the study is based, which is also different in the two countries, being more numerous in Italy than in Germany. This study focuses on two national sub-samples of informal caregivers of older people, drawn from a larger European sample whose recipients were also non-elderly care recipients. This is why the sample is quite limited.

The second limitation is represented by the convenience, not randomized, sampling strategy of participants recruited for this study, which does not allow the generalization of results to all informal caregivers in the two countries.

Another limitation is constituted by the channels chosen for the data collection, which reached only digitally literate and more highly educated people, thus excluding a priori many caregivers who are not familiar with online tools. This mirrors what is highlighted by the literature, i.e., that among older adults, the more educated have higher levels of digital skills. In fact, since complexity is an important barrier to technology adoption, people with a higher level of education seem to be more keen to overcome this type of problem [74,75]. For example, in Italy, a recent study confirmed that access to a computer and the internet at home increases with the educational level [76]. This could be the reason why in the Italian sample there are more educated individuals than in the German one (especially those having an upper secondary educational level).

Another study bias could be the under-representation within the sample of caregivers who suffered from COVID-19. This is probably due to health-related difficulties to access and fill-in the online questionnaire if caregivers were infected with the virus.

These are biases to be considered when interpreting the illustrated findings.

## 5. Conclusions

Despite the limitations highlighted above, to our knowledge this study represents one of the very few international efforts aimed at shedding light on the impact of the pandemic on one of the most invisible—albeit essential—groups of LTC providers: informal caregivers. It allows us to highlight important aspects, such as the fact that the German and Italian health and social care systems have failed to support and safeguard the health of informal caregivers during the COVID-19 outbreak.

This study also reveals that the German LTC system, commonly considered as well-structured and providing many typologies of supports, has not been able to adequately sustain informal caregivers of older people with LTC needs, especially caregivers of older care recipients with psychological/mental health issues. During the pandemic, living with older care recipients was a predictor of caregivers’ worsened health condition, as well as the discontinuity of health and social care service provision. The health of informal caregivers in Germany seems to have been more at risk than that of Italian caregivers, although the former received more services. This seems to lie in the more demanding caring context experienced by German respondents who cared for older relatives with worse health conditions as compared to the Italian ones. This finding calls for stronger home care services targeted to older people with psychological/mental diseases and respite care measures for their caregivers. Moreover, the more widespread presence of MCWs and their substantial support could have increased the resilience of the Italian LTC system during the COVID-19 pandemic, mitigating the lockdown effects, to the advantage of informal caregivers. What our study did not investigate, being outside of its scope, is whether this occurred at the detriment of MCWs’ health and well-being.

In light of these findings, further research is needed to explore more in-depth the economic, psychological and social costs of the pandemic for both informal caregivers and MCWs in the two countries and to identify policies that can better reconcile the interests of older people with LTC needs and their informal caregivers as employers, with those of MCWs, to be analysed as a crucial but largely under-investigated triad in current LTC systems.

## Figures and Tables

**Table 1:** Informal caregivers’ description and living arrangement.

	Total (*n* = 319)	Germany (*n* = 146)	Italy (*n* = 173)	*p*
**Informal Caregivers**				
Gender				0.031
Male	51 (16.04%)	15 (10.27%)	36 (20.93)	
Female	264 (83.02%)	130 (89.04%)	134 (77.9%)	
Prefer not to say	3 (0.94%)	1 (0.68%)	2 (1.16%)	
Mean age	55.4 ± 11.8	55.6 ± 11.4	55.2 ± 12.2	0.766
Educational level				0.000
Primary education	9 (2.82%)	6 (4.11%)	3 (1.73%)	
Lower secondary education	51 (15.99%)	39 (26.71%)	12 (6.94%)	
Upper secondary education	114 (35.74%)	38 (26.03%)	76 (43.93)	
Tertiary education	145 (45.45%)	63 (43.15%)	82 (47.4%)	
Caring for				0.117
Grandparent/parent/parent-in-law	226 (70.8%)	97 (66.4%)	129 (74.6%)	0.112
Spouse/Partner	59 (18.5%)	36 (24.7%)	23 (13.3%)	0.009
Other (e.g., friend, neightbour, ex-spouse/partner)	23 (7.2%)	9 (6.2%)	14 (8.1%)	0.507
Uncle/Aunt	7 (2.2%)	3 (2.1%)	4 (2.3%)	0.876
Brother/Sister or Brother/Sister-in-law	4 (1.3%)	1 (0.7%)	3 (1.7%)	0.401
Living arrangement in relation to the care recipient				0.015
In the same household	128 (40.25%)	57 (39.04%)	71 (41.28%)	
In different household but in the same building	47 (14.78%)	22 (15.07%)	25 (14.53%)	
Within walking distance	43 (13.52%)	14 (9.59%)	29 (16.86%)	
Not within walking distance but less than 30 min one-way travel	69 (21.7%)	30 (20.55%)	39 (22.67%)	
Between 30 min and one hour travelling	18 (5.66%)	13 (8.9%)	5 (2.91%)	
Between one and three hours travelling	7 (2.2%)	4 (2.74%)	3 (1.74%)	
Between three and five hours travelling	6 (1.89%)	6 (4.11%)	0 (0%)	
Mean hours of care provided per week before the COVID-19 outbreak	36.9 ± 50.9	32.4 ± 37.5	40.7 ± 59.8	0.154
Mean hours of care provided per week during the COVID-19 outbreak	45.6 ± 50.0	44.1 ± 44.8	46.8 ± 54.2	0.636
**Older care recipients**				
Gender				0.029
Male	105 (33.1%)	59 (40.7%)	46 (26.7%)	
Female	208 (65.6%)	84 (57.9%)	124 (72.1%)	
Prefer not to say	4 (1.3%)	2 (1.4%)	2 (1.2%)	
Mean age	81.2 ± 8.1	81.6 ± 8.3	80.9 ± 7.9	0.499
Physical disabilities (caused, e.g., by frailty, accident, injury, illness)	220 (73.1%)	111 (79.3%)	109 (67.7)	0.024
Psychological/mental health issues (e.g., depression, anxiety, etc.)	191 (64.5%)	93 (68.4%)	98 (61.3%)	0.201
Cognitive impairments (e.g., Alzheimer’s, dementia, etc.)	212 (70.9%)	104 (74.8%)	108 (67.5)	0.165
Neurological disability or learning difficulty (other than dementia and memory problems)	98 (33.6%)	45 (33.1%)	53 (34%)	0.873
Other chronic illnesses (e.g., diabetes, heart disease, cancer, etc.)	204 (68.7%)	102 (75.6%)	102 (63%)	0.020
Other long-term health conditions	169 (57.7%)	90 (68.7%)	79 (48.8%)	0.001

**Table 2:** Formal supports received during the COVID-19 outbreak.

	Total (*n* = 319)	Germany (*n* = 146)	Italy (*n* = 173)	*p*
Health care	152 (48.9%)	77 (53.8%)	75 (44.6%)	0.106
Social care	67 (21.9%)	42 (29.8%)	25 (15.2%)	0.002
Face-to-face help groups	52 (16.9%)	42 (29.2%)	10 (6.1%)	0.000
Counselling/information via helplines and telephone services	102 (32.8%)	53 (37.1%)	49 (29.2%)	0.139
Online support services (e.g., psychological/emotional support, etc.)	47 (15.3%)	19 (13.5%)	28 (16.8%)	0.424
Practical help (e.g., preparing meals, laundry, housework, etc.)	79 (25.5%)	50 (35%)	29 (17.4%)	0.000
Grocery/meal delivery at home	72 (23.2%)	48 (33.8%)	24 (14.2%)	0.000
Medication/drug delivery at home	93 (29.9%)	63 (44.1%)	30 (17.9%)	0.000
Transportation (e.g., to go to the General Practitioner, etc.)	62 (19.8%)	44 (30.8%)	18 (10.6%)	0.000
Respite care/Relief services	53 (17%)	35 (24.5%)	18 (10.7%)	0.001
Education and training	35 (11.3%)	16 (11.2%)	19 (11.3%)	0.973
Financial support	42 (13.5%)	24 (16.7%)	18 (10.8%)	0.130

**Table 3:** Very/extremely effective support providers during the pandemic.

Typologies of Services	Total (*n* = 319)	Germany (*n* = 146)	Italy (*n* = 173)	*p*
Public health services/professionals	42 (13.2%)	25 (17.1%)	17 (9.8%)	0.000
Public social services/professionals	28 (8.8%)	19 (13%)	9 (5.3%)	0.000
Private care services/providers	74 (23.5%)	39 (26.9%)	35 (20.6%)	0.000
Voluntary organisations	19 (6%)	2 (1.4%)	17 (9.9%)	0.000
Friends/Neighbours	75 (23.8%)	40 (27.8%)	35 (20.4%)	0.000
Migrant/private care workers	52 (16.5%)	5 (3.5%)	47 (27.7%)	0.000
Carer/patient organisation(s)	54 (17.5%)	22 (22.2%)	22 (13.7%)	0.003
General Practitioner	86 (27.3%)	42 (29%)	44 (25.9%)	0.010
Family members	128 (40.7%)	71 (48.6%)	57 (33.7%)	0.013
Church/Religious organisations	19 (6%)	11 (7.5%)	8 (4.7%)	0.029
Pharmacies	124 (39.8%)	66 (45.8%)	58 (34.7%)	0.203

**Table 4:** Difficulties faced by informal caregivers during the COVID-19 outbreak.

Types of Difficulties	Total (*n* = 319)	Germany (*n* = 146)	Italy (*n* = 173)	*p*
I have experienced difficulties for MYSELF				0.001
1-Almost always	35 (11.2%)	12 (8.4%)	23 (13.5%)	
2-Often	64 (20.4%)	20 (14%)	44 (25.9%)	
3-Sometimes	97 (31%)	42 (29.4%)	55 (32.4%)	
4-Seldom	54 (17.3%)	26 (18.2%)	28 (16.5%)	
5-Never/I have no other care recipient(s)	63 (20.1%)	43 (30.1%)	20 (20.1%)	
I feel able to look after my own health and well-being				0.000
1-Strongly agree	54 (17%)	27 (18.6%)	27 (15.6%)	
2-Quite agree	107 (33.6%)	34 (23.4%)	73 (42.2%)	
3-Undecided	87 (27.4%)	38 (26.2%)	49 (28.3%)	
4-Quite disagree	51 (16%)	30 (20.7%)	21 (12.1%)	
5-Strongly disagree	19 (6%)	16 (11%)	3 (1.7%)	
I feel overwhelmed due to the COVID-19 outbreak				0.001
1-Strongly agree	66 ( 20.8%)	26 (17.9%)	40 (23.3%)	
2-Quite agree	83 (26.2%)	27 (18.6%)	56 (32.6%)	
3-Undecided	87 (27.4%)	55 (37.9%)	32 (18.6%)	
4-Quite disagree	60 (18.9%)	28 (19.3%)	32 (18.6%)	
5-Strongly disagree	21 (6.6%)	9 (6.2%)	12 (7%)	

**Table 5:** Overall informal caregivers’ perceived health status and worsening predictors by country.

Predictors	Worsened Overall Health Status OR (95%CI)					
	Germany (*n* = 145)			Italy (*n* = 172)		
	Improved/Not Changed Health (*n* = 60)	Worsened Health (*n* = 85)	*p*	Improved/Not Changed Health (*n* = 93)	Worsened Health (*n* = 79)	*p*
**Caregivers**						
Age	53.2 ± 13.1	57.0 ± 9.5	0.050	55.2 ± 12.1	54.8 ± 12.0	0.814
Gender (Female)	53 (88.3%)	76 (89.4%)	0.838	69 (75.0%)	65 (82.3%)	0.249
Educational level			0.528			0.824
Primary education	2 (3.3%)	4 (4.7%)		1 (1.1%)	2 (2.5%)	
Lower secondary education	14 (23.3%)	25 (29.4%)		6 (6.5%)	6 (7.6%)	
Upper secondary education	14 (23.3%)	24 (28.2%)		40 (43.0%)	36 (45.6%)	
Tertiary education	30 (50.0%)	32 (37.6%)		46 (49.5%)	35 (44.3)	
Caregiving (Difference post–pre in hours of care)	8.8 ± 20.9	13.9 ± 17.1	0.118	4.4 ± 31.4	10.7 ± 25.1	0.157
Service provision (Continuity)	41 (69.5%)	52 (61.9%)	0.349	66 (71.7%)	58 (74.4%)	0.702
Formal social and health care services provision (Discontinuity)	46 (78.0%)	71 (83.5%)	0.400	72 (79.1%)	71 (91.0%)	**0.032**
Formal support effectiveness (at least 1 very/extremely)	46 (76.7%)	58 (68.2%)	0.267	55 (59.1%)	51 (64.6%)	0.467
Informal support effectiveness (at least 1 very/extremely)	42 (70.0%)	42 (49.4%)	**0.013**	58 (62.4%)	44 (56.4%)	0.429
Living condition			0.077			0.072
Co-habiting	25 (41.7%)	53 (62.4%)		45 (48.4%)	50 (64.1%)	
Walking distance	6 (10.0%)	8 (9.4%)		20 (21.5%)	9 (11.5%)	
Within 1 h travel	23 (38.3%)	20 (23.5%)		25 (26.9%)	19 (24.4%)	
More than 1 h travel	6 (10.0%)	4 (4.7%)		3 (3.2%)	0 (0.0%)	
Caregiver Infection (“I have personally been infected”)	3 (5.8%)	9 (11.4%)	0.275	7 (8.6%)	14 (18.7%)	0.067
**Older care recipient**						
Psychological/mental health issues (e.g., depression, anxiety, etc.)	29 (52.7%)	64 (79.0%)	**0.001**	45 (52.9%)	52 (70.3%)	**0.025**
Neurological disability or learning difficulty (other than dementia and memory problems)	14 (25.0%)	31 (38.8%)	0.094	21 (25.6%)	31 (42.5%)	**0.027**

“Bold” higlight the statistically significant differences.

**Table 6:** Predictors of informal caregivers’ overall health status worsening by country.

	Worsened Overall Health Status OR (95%CI)					
	Germany			Italy		
Predictors	Crude Model	Age and Gender Adjusted Model	Fully adjusted Model *	Crude Model	Age and Gender Adjusted Model	Fully Adjusted Model **
**Caregivers**						
Age	1.03 (1.00–1.06)	1.03 (1.00–1.06)	1.03 (0.99–1.06)	1.00 (0.97–1.02)	1.00 (0.97–1.03)	1.00 (0.97–1.03)
Gender (Female)	1.11 (0.39–3.18)	1.23 (0.42–3.60)	1.15 (0.37–3.60)	1.55 (0.73–3.26)	1.54 (0.72–3.32)	1.48 (0.68–3.24)
Educational level	0.531	0.599		0.832	0.780	
Primary education	-	-		-	-	
Lower secondary education	0.89 (0.14–5.50)	1.15 (0.18–7.32)		0.50 (0.03–7.10)	0.38 (0.02–5.95)	
Upper secondary education	0.86 (0.14–5.29)	1.29 (0.20–8.43)		0.45 (0.04–5.17)	0.30 (0.02–3.99)	
Tertiary education	0.53 (0.09–3.13)	0.75 (0.12–4.65)		0.38 (0.03–4.37)	0.28 (0.02–3.72)	
Caregiving (Difference post–pre in hours of care)	1.02 (0.99–1.04)	1.02 (1.00–1.04)		1.01 (1.00–1.02)	1.01 (0.99–1.02)	
Service provision (Continuity)	0.71 (0.35–1.45)	0.81 (0.39–1.69)		1.14 (0.58–2.26)	1.15 (0.57–2.33)	
Formal social and health service provision (Discontinuity)	1.43 (0.62–3.32)	1.66 (0.70–3.94)		2.68 (1.06–6.76)	2.54 (0.99–6.48)	**2.54** (1.00–6.48)
Formal support effectiveness (at least 1 very/extremely)	0.65 (0.31–1.39)	0.63 (0.29–1.37)		1.26 (0.68–2.34)	1.31 (0.69–2.46)	
Informal support effectiveness (at least 1 very/extremely)	0.42 (0.21–0.84)	0.45 (0.22–0.91)	**0.45** (0.23–0.99)	0.78 (0.42–1.44)	0.82 (0.43–1.54)	
Living condition	0.083	0.075		0.230	0.263	
reference: *co-habiting*	-	-		-	-	
Walking distance	0.63 (0.20–2.01)	0.69 (0.21–2.26)		0.40 (0.17–0.98)	0.40 (0.16–0.99)	
Within 1 h travel	0.41 (0.19–0.88)	0.39 (0.18–0.85)		0.68 (0.33–1.40)	0.76 (0.36–1.61)	
More than 1 h travel	0.31 (0.08–1.21)	0.32 (0.08–1.26)		-	-	
Caregiver Infection (“I have personally been infected”)	2.10 (0.54–8.16)	1.81 (0.46–7.20)		2.43 (0.92–6.39)	2.64 (0.94–7.45)	
**Older care recipients**						
Psychological/mental health issues (e.g., depression, anxiety, etc.)	3.37 (1.59–7.16)	3.14 (1.45–6.77)	**3.01** (1.38–6.56)	2.10 (1.09–4.05)	1.99 (1.01–3.91)	
Neurological disability or learning difficulty (other than dementia and memory problems)	1.89 (0.89–4.03)	1.85 (0.85–4.02)		2.14 (1.09–4.23)	1.86 (0.93–3.73)	

Significant ORs in bold. * Cox and Snell R-square is 0.110. ** Cox and Snell R-square is 0.031.

## Data Availability

The study cross-national data gathered through the online survey are fully available in the report available online at https://eurocarers.org/wp-content/uploads/2021/05/EUC-Covid-study-report-2021.pdf (assessed on 18 December 2021).

## References

[1] EUROSTAT (2021). Population Structure and Ageing. https://ec.europa.eu/eurostat/statistics-explained/index.php?title=Population_structure_and_ageing#The_share_of_elderly_people_continues_to_increase.

[2] Laenen T., Rossetti F., van Oorschot W. (2019). Why deservingness theory needs qualitative research: Comparing focus group discussions on social welfare in three welfare regimes. Int. J. Comp. Sociol..

[3] Geraedts M., Heller G.V., Harrington C.A. (2000). Germany’s Long-Term-Care Insurance: Putting a Social Insurance Model into Practice. Milbank Q..

[4] Theobald H., Luppi M. (2018). Elderly care in changing societies: Concurrences in divergent care regimes–A comparison of Germany, Sweden and Italy. Curr. Sociol..

[5] Heiber A. (2020). System & Praxis.

[6] Bahnsen L., Raffelhüschen B. (2019). Zur Reform der Pflegeversicherung [On the Reform of Long-Term Care Insurance].

[7] Esping-Andersen G. (1999). Social Foundation of Postindustrial Economies.

[8] Rotolo A., Fosti G., Notarnicola E. (2014). Modelli istituzionali e percorsi degli utenti. Il Welfare e la Long Term Care in Europa [Welfare System and Long-Term Care in Europe].

[9] Costa G. (2013). Private Assistants in the Italian Care System: Facts and Policies. Obs. Soc. Br..

[10] Courbage C., Montoliu-Montes G., Wagner J. (2020). The effect of Long-Term Care public benefits and insurance on informal care from outside the household: Empirical evidence from Italy and Spain. Eur. J. Health Econ..

[11] Barbabella F., Poli A., Chiatti C., Pelliccia L., Pesaresi F. (2017). La bussola di NNA: Lo stato dell’arte basato sui dati. L’assistenza Agli Anziani Non Autosufficienti in Italia. 6 Rapporto 2017/2018. Il Tempo Delle Risposte [Assistance to Non Self-Sufficient Elderly People in Italy. 6th Report 2017/2018. Time for Answers].

[12] Berloto S., Notarnicola E., Notarnicola E., Fosti G. (2019). La prospettiva dei policy-makers: Quali temi e innovazioni stanno promuovendo le regioni?. Il Futuro del Settore LTC: Prospettive dai Servizi, dai Gestori e Dalle Policy Regionali 2 Rapporto Osservatorio Long Term Care [The Future of the LTC: Perspectives from Services, Providers and Regional Policies. 2nd Report LTC Observatory].

[13] Gori C., Guber E. (2021). L’indennità di accompagnamento. L’assistenza Agli Anziani Non-Autosufficienti in Italia, 7 raporto 2020–2021. Punto di Non Ritorno [The Assistance of Non Self-Sufficient Older People in Italy, 7th Report 2020-2021. Point of Non-Returning].

[14] Frank J., Babitsch B. (2018). Kompression oder Expansion der Morbidität in der ambulanten Versorgung? [Compression or expansion of morbidity in outpatient care?]. Z. Gerontol. Geriatr..

[15] Destatis (2020). Pflegestatistik 2019 [Care Statistics 2019].

[16] Döbele M., Becker U. (2016). Pflegeversicherung, in Ambulante Pflege von A bis Z.

[17] Schütte W. (2009). Das Leistungskonzept der Pflegeversicherung im Reformprozess—Angehörigenpflege, Pflegegeld und, Neues Verwaltungsrecht. Sozialgerichtsbarkeit.

[18] Li J. (2019). Study and Comparison of Elderly Care System in Germany and China. Ph.D. Thesis.

[19] ISTAT-Istituto Nazionale di Statistica-Italian [National Institute of Statistics] (2020). Censimento Permanente Della Popolazione e Abitazioni [Permanent Census of the Population and Housing].

[20] ISTAT-Istituto Nazionale di Statistica-Italian [National Institute of Statistics] (2019). Le Condizioni di Salute Della Popolazione Anziana in Italia. Anno 2019 [Health Condition of Older Population in Italy. Year 2019].

[21] CERGAS-Centro Ricerca sulla Gestione dell’Assistenza [Research Center on Care Management] (2019). Rapporto OASI [OASI Report].

[22] European Commission and Social Protection Committee (2021). Long-Term Care Report. Trends, Challenges and Opportunities in an Ageing Society.

[23] European Commission (2018). Informal Care in Europe. Exploring Formalisation, Availability and Quality.

[24] Tur-Sinai A., Teti A., Rommel A., Hlebec V., Lamura G. (2020). How many older informal caregivers are there in Europe? Comparison of estimates of their prevalence from three european surveys. IJERPH.

[25] European Commission and Social Protection Committee (2021). The European Pillar of Social Rights in 20 principles.

[26] Horn V., Schweppe C., Böcker A.G.M., Bruquetas Callejo M.M. (2019). Live-in migrant care worker arrangements in Germany and the Netherlands. Motivations and justifications in family decision-making. Int. J. Ageing Later Life.

[27] Steiner J., Prieler V., Leiblfinger M., Benazha A. (2019). Völlig legal!? Rechtliche Rahmung und Legalitätsnarrative in der 24h-Betreuung in Deutschland, Österreich und der Schweiz. Osterr. Z. fur Soziol.

[28] INPS-Istituto Nazionale per la Previdenza Sociale [Italian National Institute for Social Protection] (2021). Osservatorio sui Lavori Domestici [Italy’s Domestic Workers Observatory].

[29] Del-Pino-Casado R., Rodrıguez Cardosa M., Lopez-Martınez C., Orgeta V. (2019). The association between subjective caregiver burden and depressive symptoms in carers of older relatives: A systematic review and meta-analysis. PLoS ONE.

[30] Hooker K., Bowman S.R., Coehlo D.P., Lim S.R., Kaye J., Guariglia R., Li F. (2002). Behavioral change in persons with dementia: Relationships with mental and physical health of caregivers. J. Gerontol. B Psychol. Sci. Soc. Sci..

[31] Bom J., Bakx P., Schut F., van Doorslaer E. (2019). The Impact of Informal Caregiving for Older Adults on the Health of Various Types of Caregivers: A Systematic Review. Gerontologist.

[32] Pinquart M., Sörensen S. (2011). Spouses, Adult Children, and Children-in-Law as Caregivers of Older Adults: A Meta-Analytic Comparison. Psychol. Aging.

[33] Verbakel E. (2018). How to Understand Informal Caregiving Patterns in Europe? The Role of Formal Long-Term Care Provisions and Family Care Norms. Scand. J. Public Health.

[34] Barbosa F., Voss G., Delerue Matos A. (2020). Health impact of providing informal care in Portugal. BMC Geriatr..

[35] Santini S., Andersson G., Lamura G. (2016). Impact of incontinence on the quality of life of caregivers of older persons with incontinence: A qualitative study in four European countries. Arch Gerontol. Geriatr..

[36] Mohanty I., Niyonsenga T. (2019). A longitudinal analysis of mental and general health status of informal carers in Australia. BMC Public Health.

[37] Litwin H., Stoeckel K.J., Roll A. (2014). Relationship Status and Depressive Symptoms among Older Co-Resident Caregivers. Aging Ment. Health.

[38] Censis-AIMA (2016). L’impatto Economico e Sociale Della Malattia di Alzheimer: Rifare il Punto Dopo 16 Anni [The Economic and Social Impact of Alzheimer’s Disease: Take Stock after 16 Years].

[39] Ikeda S. (2017). Supporting working carers’ job continuation in Japan: Prolonged care at home in the most aged society. Int. J. Care Caring.

[40] Socci M., Principi A., Di Rosa M., Carney P., Chiatti C., Lattanzio F. (2021). on behalf of the UP-TECH Research Group. Impact of working situation on mental and physical health for informal caregivers of older people with Alzheimer’s disease in Italy. Results from the UP-TECH longitudinal study. Aging Ment. Health.

[41] Greenwood N., Mezey G., Smith R. (2018). Social exclusion in adult informal carers: A systematic narrative review of the experiences of informal carers of people with dementia and mental illness. Maturitas.

[42] Sumner A., Hoy C., Ortiz-Juarez E. (2020). Estimates of the Impact of COVID-19 on Global Poverty.

[43] Hale T., Webster S., Petherick A., Phillips T., Kira B. (2020). Oxford COVID-19 Government Response Tracker.

[44] CIRCLE (2020). Caring and COVID-19. Loneliness and Use of Services.

[45] Lightfoot E., Moone R.P. (2020). Caregiving in Times of Uncertainty: Helping Adult Children of Aging Parents Find Support during the COVID-19 Outbreak. J. Geront. Soc. Work.

[46] Carers UK (2020). Caring behind Closed Doors: Forgotten Families in the Coronavirus Outbreak.

[47] Giebel C., Cannon J., Hanna K., Butchard S., Eley R., Gaughan A., Komuravelli A., Shenton J., Callaghan S., Tetlow H. (2021). Impact of COVID-19 related social support service closures on people with dementia and unpaid carers: A qualitative study. Aging Ment. Health.

[48] Gräler L., Bremmerset L., van Excel J., Bakx P., van Bochove M. Informal care in times of public health crisis: Objective burden, subjective burden and quality of life of caregivers in the Netherlands during the COVID-19 pandemic. Proceedings of the Presented at the VID Conference ‘Demographic Aspects of the COVID-19 Pandemic and Its Consequences’.

[49] Lorenz-Dant K. (2020). Germany and the COVID-19 Long-Term Care Situation. LTC Covid.

[50] Maccora J., Ee N., Hosking D., McCallum J. (2020). Who Cares? Older Australians Do.

[51] Phillips D., Paul G., Fahy M., Dowling-Hetherington L., Kroll T., Moloney B., Duffy C., Fealy G., Lafferty A. (2020). The invisible workforce during the COVID-19 pandemic: Family carers at the frontline. HRB Open Res.

[52] Rothgang H., Wolf-Ostermann K., Domhoff D., Franziska H., Heß M., Kalwitzki T., Ratz K., Schmidt A., Seibert K., Stolle C. (2020). How Covid-19 Has Affected Informal Caregivers and Their Lives in Germany.

[53] Eurocarers/IRCCS-INRCA (2021). Impact of the Covid-19 Outbreak on Informal Carers across Europe. Final Report.

[54] Giebel C., Pulford D., Cooper C., Lord K., Shenton J., Cannon J., Shaw L., Tetlow H., Limbert S., Callaghan S. (2021). COVID-19-related social support service closures and mental well-being in older adults and those affected by dementia: A UK longitudinal survey. BMJ Open.

[55] Vahia I.V., Blazer D.G., Smith G.S., Karp J.F., Steffens D.C., Forester B.P., Reynolds C.F. (2020). COVID-19, mental health and aging: A need for new knowledge to bridge science and service. Am. J. Geriatr. Psychiatry.

[56] Kostyál L.Á., Széman Z., Almási V.E., Fabbietti P., Quattrini S., Socci M., Lamura G., Gagliardi C. (2021). Impact of the COVID-19 Pandemic on Family Carers of Older People Living with Dementia in Italy and Hungary. Sustainability.

[57] Schmidt A.E., Leichsenring K., Stafinger H., Litwin C., Bauer A. (2020). The Impact of COVID19 on Users and Providers of Long-Term Care Services in Austria. Country Report in LTC.

[58] Leiblfinger M., Prieler V., Schwiter K., Steiner J., Benazha A., Lutz H. (2020). Impact of the COVID-19 Pandemic on Live-in Care Workers in Germany, Austria, and Switzerland.

[59] Kent E.E., Ornstein K.A., Dionne-Odom J.N. (2020). The family caregiving crisis meets an actual pandemic. J. Pain Symptom. Manag..

[60] Onwumere J. (2021). Informal carers in severe mental health conditions: Issues raised by the United Kingdom SARS-CoV-2 (COVID-19) Pandemic. Int. J. Soc. Psychiatry.

[61] Carers Trust (2020). My Future, My Feelings, My Family: How Coronavirus is Afecting Young Carers and Young Adult Carers, and What They Want You to Do Next.

[62] Rodrigues R., Simmons C., Schmidt A.E., Steober N. (2021). Care in times of COVID-19: The impact of the pandemic on informal caregiving in Austria. Eur. J. Ageing.

[63] Tur-Sinai A., Bentur N., Fabbietti P., Lamura G. (2021). Impact of the Outbreak of the COVID-19 Pandemic on Formal and Informal Care of Community-Dwelling Older Adults: Cross-National Clustering of Empirical Evidence from 23 Countries. Sustainability.

[64] Mah J.C., Stevens S.J., Keefe J.M., Rockwood K., Andrew M.K. (2021). Social factors influencing utilization of home care in community-dwelling older adults: A scoping review. BMC Geriatr..

[65] European Commission (2021). European Pillars of Social Rights Action Plan.

[66] Van Hooren F. (2020). COVID-19, Migrant Workers and the Resilience of Social Care in Europe.

[67] (2020). Decree of the President of the Council of Ministers-DPCM 08.03.2020, Moving Restrictions in Several Italian Regions, Rome, Italy. https://www.gazzettaufficiale.it/eli/id/2020/03/08/20A01522/sg.

[68] (2020). Decree of the President of the Council of Ministers-DPCM 09.03.2020, Moving Restrictions in the Whole National Territory: Italy Red Zone, Rome, Italy. https://www.gazzettaufficiale.it/eli/id/2020/03/09/20A01558/sg.

[69] Wang H., Li T., Barbarino P., Gauthier S., Brodaty H., Molinuevo J.L., Xie H., Sun Y., Yu E., Tang Y. (2020). Dementia care during COVID-19. Lancet.

[70] Lloyd-Sherlock P., Ebrahim S., Geffen L., McKee M. (2020). Bearing the brunt of covid-19: Older people in low and middle income countries. BMJ.

[71] OECD (2020). Who Cares? Attracting and Retaining Care Workers for the Elderly.

[72] Fasani F., Mazza J. (2020). Immigrant Key-Workers: Their Contribution to Europe’s COVID-19 Response.

[73] European Commission (2020). JRC Technical Report. A Vulnerable Work-Force: Migrant Workers in the COVID-19 Pandemic.

[74] Rogers E. (2005). Diffusion of Innovations.

[75] Zhao H., Kim S., Suh T., Du J. (2007). Social institutional explanations of global internet diffusion: A cross-country analysis. J. Glob. Inf. Manag..

[76] Di Pietro G. (2021). Changes in Italy’s education-related digital divide. Econ. Aff..

